# We need to talk about validity – A commentary on “Six solutions for more reliable infant research” from the viewpoint of an early executive functions researcher

**DOI:** 10.1002/icd.2352

**Published:** 2022-06-07

**Authors:** Karla Holmboe

**Affiliations:** ^1^ School of Psychological Science University of Bristol Bristol UK

**Keywords:** construct validity, executive functions, infancy, methodology, reliability

## Abstract

In their methodological article, “Six solutions for more reliable infant research”, Byers‐Heinlein, Bergmann and Savalei (2021) present compelling arguments for why developmental researchers should report and consider measures of reliability more frequently in their work. They also provide useful guidance on solutions to this “reliability crisis”. In this commentary, I highlight a further methodological aspect that I think is key to successful and robust infancy research, that of construct validity. I also discuss recent reliability data from my own research on early executive function development, analyses which were directly inspired by the target article.

**Highlights:**

- Considering measurement reliability and effect sizes is important for robust infant research and for optimising infant tasks to measure group‐level effects or individual differences.
- Construct validity – making sure that we measure what we think we are measuring – is also important.
- A robust effect at the group‐level may not always restrict reliability – it depends on the amount of true variation between infants.

“Six solutions for more reliable infant research”, by Byers‐Heinlein et al. (2021), immediately caught my attention when I first came across the preprint on Twitter (Byers‐Heinlein, 2021). I consider it an important paper for the field of infancy research because it highlights key issues that we need to address, as individuals and as a field, to make our research robust, reliable, and also feasible given the practical challenges that we face as infant researchers in terms of recruitment and data attrition. The preprint had a direct impact on a paper I was revising at the time (Holmboe et al., 2021), by propelling me to include analyses of within‐session reliability (some of which I summarise below), and I plan to continue incorporating the “six solutions” in my research.

I decided to write this commentary, not because I have any major points of critique or see any substantial weaknesses in Byers‐Heinlein et al.'s (2021) arguments – in fact, I agree with most of the issues that the authors raise, as well as their concrete recommendations. Instead, I hope I can contribute some additional thoughts and considerations from the viewpoint of an early executive functions researcher, which will further stimulate this interesting discussion.

Validity and reliability are the Yin and Yang of any undergraduate textbook on research methods. At least within a quantitative tradition, we need to be able both to measure the construct of interest and to do so reliably. Byers‐Heinlein et al. (2021) rightly highlight the many issues with reliability that infancy research has had over the years, such as issues with statistical power, data attrition and the optimisation of tasks for measuring group‐level constructs versus individual differences. Their specific suggestions as to how we can improve measurement in infant research (Solutions 2 and 3) focus primarily on obtaining and using information about effect sizes and reliability. In this commentary, I would like to add a few thoughts on validity, in particular construct validity, which I think are complimentary to the important discussion the authors raise about reliability.

In addition to making sure that we measure something reliable, we also need to know *what* we are measuring. In the adult metascience literature, validity has recently been highlighted as an important part of the wider “credibility revolution” (Flake & Fried, 2020; Vazire et al., 2022). For example, Flake and Fried (2020) emphasise that thorough and transparent reporting of *measure use* (including the entire process from conceptualisation to calculation of specific indices) is essential to ensure that study claims are valid. Similarly, Vazire et al. (2022) emphasise that it is not enough for psychological research to be replicable, we also need to make sure that research is credible in a wider sense. To achieve this, it is necessary to consider the “four validities” (construct validity, internal validity, external validity and statistical‐conclusion validity), which Vazire et al. (2022) describe alongside striking examples of how psychological research can appear rigorous and methodologically sound but nevertheless not support the constructs or conclusions that researchers put forward.

When it comes to the key task of defining psychological constructs, both Flake and Fried (2020) and Vazire et al. (2022) point to poor conceptual clarity, and the interlinked issue of poor operationalisation into specific measures, as issues that hamper validity. I believe that similar issues riddle infant research. My own field, early executive function development, is a case in point. Executive functions, often defined as the overlapping domains of working memory, inhibitory control and cognitive flexibility, are considered higher‐order cognitive functions (Diamond, 2013; Miyake et al., 2000), and are challenging even for adults, which makes investigating them in infants far from trivial. My research over the last 5 years has focused on developing new tasks to measure executive functions in infancy and toddlerhood, with the broader aim of being able to track individual differences in these skills over time. To do this, we have often had to go back to the drawing board and really think about how we can achieve validity of our new measures. How can we know that a non‐verbal infant is *actually* representing an object in working memory? How can we get infants to inhibit a prepotent action, and can we be sure that this is in fact what they are doing (or not doing) when they are performing the task? Whereas reliability can be tested statistically with relative ease, this kind of *construct validity* (i.e., are we really measuring what we are trying to measure?) is much harder to assess. We can design our task conditions to make it more *likely* that we are measuring the ability we are interested in, we can add control conditions to rule out the most compelling alternative explanations, and we can correlate with more established “gold standard” tasks (if they exist) to try to confirm that our new task is measuring the same “thing”, but fundamentally, establishing construct validity is a slow and arduous process which takes years.

Too often, I think, developmental researchers pick out a task to use from the literature for the mundane reason that it has been published and somebody (possibly highly‐regarded) has used it before. Construct validation in the traditional sense of a full psychometric evaluation, cross‐validation with gold standard measures, establishment of convergent and divergent validity and factor analysis is rarely undertaken in infant research. There are good reasons for this – for example, factor analysis requires multiple tasks and a large number of participants, which can be challenging to achieve with infants. However, I do think it is important to continue exploring, and sometimes even challenging, *what* we are measuring. If we do not stop and reflect (and conduct research!) on this, we may end up using tasks for years that do not actually measure what we think they measure, and this clearly has important implications for the broader credibility of infant research (as it does for adult research, cf. Vazire et al., 2022). Reliability and validity therefore need to go hand in hand – we need to both understand what we are measuring, and we need to show that we are measuring this function or trait reliably. I realise that the authors of “Six solutions for reliable infant research” do not argue that validity is not important, so I am simply highlighting this as another key aspect to consider when promoting robust and meaningful infant research.

Another point I would like to comment on is the interesting statistical modelling that Byers‐Heinlein et al. (2021) present (Figure 1 in the target article), indicating that tasks that produce robust experimental effects at the group‐level tend to have low reliability, at least when *true variability* is low. There is a growing literature in adults confirming this picture (Enkavi et al., 2019; Hedge et al., 2018; Schuch et al., 2021). This is a very important point for individual differences researchers, because we want to measure those differences!. However, based on recent work with our newly developed Early Childhood Inhibitory Touchscreen Task (ECITT), I would argue that this might not be as severe a problem in research with young children because individual differences are often abundant at this age, at least in multi‐trial paradigms. Holmboe et al. (2021) found both substantial condition effects *and* high internal consistency (most alphas > 0.70) in toddlers aged between 18 and 24 months (for full analyses and the trial‐level data, see: https://osf.io/ytfdp/). Interestingly, the only age group where internal consistency dropped to an unacceptable level was at 30 months, where performance approached ceiling and the condition effect was non‐significant. The low internal consistency at this age was therefore likely due to a restricted range of scores. This emphasises the need for well‐calibrated assessments at each age, as discussed extensively in Holmboe et al. (2021). We have also assessed 1‐week test–retest reliability of the ECITT in 10‐month‐old infants. Hendry et al. (2021) confirmed that test–retest reliability at this age was indeed modest (but significant) at *r* = 0.30, however this increased (in a separate sample) to *r* = 0.48 when more trials were administered at retest (Fiske et al., 2022), confirming that adding more trials may indeed improve reliability as per Solution 4 in Byers‐Heinlein et al. (2021). Longitudinal stability (which reflects trait stability in addition to measurement reliability) between 18 and 21 months and between 21 and 24 months was moderate‐to‐high (both *r*s > 0.6) (Holmboe et al., 2021).

I believe that these results indicate that, although test–retest reliability estimates from my own research leave room for improvement at the youngest ages, for example by implementing some of Byers‐Heinlein et al.'s (2021) suggestions, within‐session reliability can in fact be reasonably good, even in the presence of substantial condition effects. I guess it comes down to whether there is indeed a large amount of true variation between individual infants on a specific task, which in most cases is a question we can address empirically ‐ certainly, we can check whether there appears to be a *lack of* variation.

Despite the many challenges of testing squirmy babies, I think the future of infant research is bright. Equipped with important new analytical tools and the suggested solutions to the “reliability crisis” proposed by Byers‐Heinlein et al. (2021), as well as an eager eye on the construct validity of our measures, I believe we have much still to discover about infant cognition.

## AUTHOR CONTRIBUTIONS

**Karla Holmboe:** Conceptualization; writing – original draft; writing – review and editing.

### PEER REVIEW

The peer review history for this article is available at https://publons.com/publon/10.1002/icd.2352.

## Data Availability

The data pertaining to Holmboe et al. (2021) is available on OSF (https://osf.io/ytfdp/). No other data were used in this commentary.
